# Comprehensive Evaluation of the Flame-Retardant, Rheological, and Durability Performance of Fast-Melting Warm-Mix Composite Modified Asphalt Binders

**DOI:** 10.3390/ma19153325

**Published:** 2026-08-05

**Authors:** Ming Lv, Yongkang Fu, Jinchao Yue, Zikai Xu, Shenyuan Wang, Yangming Gao, Chao Zhang

**Affiliations:** 1 Henan Central Construction Engineering Co., Ltd., Zhengzhou 450001, China; 2 Poly Changda Overseas Engineering Co., Ltd., Guangzhou 510620, China; 3 School of Water Conservancy and Transportation, Zhengzhou University, Zhengzhou 450001, China; 4 School of Highway, Chang’an University, Xi’an 710064, China; 5 Built Environment and Sustainable Technologies (BEST) Research Institute, Liverpool John Moores University, Liverpool L3 3AF, UK

**Keywords:** warm-mix asphalt, SBS-modified asphalt, flame-retardant asphalt binder, aging behavior, microscopic characterization

## Abstract

Improving the flame-retardant performance of asphalt is particularly important for tunnel pavements, where confined environments can intensify fire hazards and smoke accumulation. This study prepared different modified asphalt binders to investigate their flame-retardant performance and rheological properties. The limiting oxygen index and smoke density rating were first used to evaluate the flame-retardant and smoke-suppression performance. Frequency sweep tests were then conducted to analyze the rheological behavior, aging characteristics, and low-temperature cracking resistance of the binders. Finally, microscopic tests were performed to reveal the thermal decomposition behavior and modification mechanism. Results showed that the incorporation of FR02 increased the limiting oxygen index of the warm-mix modified binders by more than 47%. Among the investigated binders, 13% fast-melting warm-mix flame-retardant composite modifier (SBS-WZ) exhibited the highest limiting oxygen index of 30.95% and the lowest smoke density rating of 57.73, indicating the best experimentally measured flame-retardant and smoke-suppression performance. At a reduced frequency of approximately 10^−2^ rad/s, the unaged 13%SBS-WZ binder exhibited a complex modulus of approximately 2.0 × 10^5^ Pa, nearly one order of magnitude higher than those of the conventional 4%SBS- and 4%fast-melting SBS modifier (SBS-T), while its phase angle was approximately 6–10° lower. At −24 °C, the creep stiffness and creep rate of the unaged 13%SBS-WZ binder were approximately 654 MPa and 0.246, respectively. After Pressure Aging Vessel (PAV) ageing, these values changed to approximately 720 MPa and 0.237. Moreover, the onset decomposition temperature of 13%SBS-WZ was 392.1 °C, which was 17.2 °C higher than that of 4%SBS-T. Together with its higher residual mass, this result suggests enhanced thermal stability and residue-forming potential, which may partly explain the measured improvements in flame-retardant and smoke-suppression performance. However, the increased complex modulus and reduced creep rate indicate a concurrent loss of low-temperature flexibility. The findings can provide theoretical guidance and technical support for the application of this material in tunnel asphalt pavements.

## 1. Introduction

Asphalt pavement has been widely used in road tunnels because of its good driving comfort, low noise, convenient maintenance, and favorable waterproofing performance [1,2,3]. However, tunnels are semi-enclosed traffic environments. Once a fire occurs, heat and smoke are difficult to dissipate rapidly, which may accelerate temperature rise, reduce visibility, and increase evacuation risks [4,5]. Upon heating, light components first volatilize, while heavier fractions undergo thermal decomposition and generate combustible hydrocarbons, carbon monoxide, and other volatile products. Once the concentration and temperature of these products become sufficient, gas-phase oxidation sustains flaming combustion. Meanwhile, oxidation and condensation reactions occur in the remaining asphalt phase, producing carbonaceous residues. Flame retardants may interfere with this process through endothermic decomposition, dilution of combustible gases, inhibition of heat and oxygen transfer, and promotion of a thermally stable residue layer. Recent experimental and numerical studies have further demonstrated that asphalt pavements are not merely passive surfaces during tunnel fires. Combustion of asphalt binders can provide an additional fuel source, increasing the heat release rate, smoke production, and emission of toxic gases, thereby accelerating fire development and deteriorating tunnel visibility and evacuation conditions [6,7]. Therefore, improving the flame-retardant and smoke-suppression performance of asphalt materials is essential for tunnel pavement applications.

Warm-mix asphalt technology has attracted increasing attention in tunnel pavement construction because it can reduce mixing and compaction temperatures, decrease energy consumption, and lower construction emissions [8,9]. Wasiuddin et al. [10] investigated the effects of Sasobit and Aspha-Min on the rheological properties of asphalt binders and reported that Sasobit could effectively reduce the mixing temperature while maintaining acceptable binder performance. Li et al. [9] studied the compaction temperatures of Sasobit-produced warm-mix asphalt mixtures modified with SBS and found that Sasobit improved the workability of SBS-modified asphalt mixtures; field test sections in tunnels further confirmed the feasibility of the recommended construction temperatures. Huo et al. [11] developed a warm-mix fast-melting SBS modifier and showed that fast-melting technology could improve the viscoelastic behavior and thermal stability of SBS-modified asphalt, while also changing its microstructural characteristics. More recently, Yang et al. [12] prepared a direct-to-plant warm-mixing flame-retardant SBS composite modified asphalt and demonstrated that the combination of warm-mix and flame-retardant technologies could improve flame retardancy and road performance. Previous studies generally agree that SBS improves binder elasticity, deformation recovery, and rutting resistance. However, these benefits depend strongly on the SBS type, dosage, base-asphalt composition, preparation conditions, and storage history, and improved rheological performance does not necessarily guarantee satisfactory phase stability [13,14]. Sasobit is consistently reported to reduce viscosity and improve workability at elevated temperatures, but its crystallization after cooling may increase binder stiffness and weaken low-temperature stress relaxation [9,10]. Similarly, flame retardants generally improve the limiting oxygen index, delay thermal decomposition, and suppress smoke generation, whereas relatively high dosages may adversely affect binder flexibility and low-temperature cracking resistance. These findings indicate that flame retardancy, workability, rheology, aging sensitivity, and cracking resistance should be evaluated together rather than independently.

The combined behavior of SBS, Sasobit, and Flame retardant (FR02) is governed by both complementary and competing effects. SBS absorbs part of the lighter asphalt fractions and forms a swollen polymer-rich phase, thereby improving elasticity, deformation recovery, and structural stability. Sasobit reduces binder viscosity above its melting range and facilitates mixing and compaction. After cooling, however, the crystallization of Sasobit may increase binder stiffness and restrict molecular mobility, which can weaken low-temperature stress-relaxation capacity. FR02 is incorporated primarily to improve flame retardancy and smoke suppression through heat absorption, dilution of combustible volatiles, and promotion of thermally stable residues. Nevertheless, its particulate nature and relatively high dosage may increase stiffness and interfere with the flexibility provided by the SBS-modified phase. However, previous studies have generally investigated warm-mix additives, fast-melting SBS modifiers, and flame-retardant components separately or through conventional multi-component blending. Research on fast-melting SBS has mainly focused on workability, dispersion, viscoelastic behavior, and thermal stability, without simultaneously examining flame retardancy and smoke suppression. Conversely, existing warm-mix flame-retardant studies have commonly used conventional SBS binders with separately added warm-mix agents and flame retardants, rather than an integrated fast-melting warm-mix flame-retardant composite modifier. Consequently, the combined effects of such an integrated modifier on limiting oxygen index, smoke density rating, ageing-dependent rheological behavior, and low-temperature cracking resistance remain insufficiently understood. Systematic comparisons between the integrated composite modifier and separately blended SBS/SBS-T–Sasobit–FR02 systems prepared and tested under identical conditions are also limited [15,16]. Therefore, further investigation is required to understand the interaction among these components and their influence on the comprehensive performance of modified asphalt binders.

In recent years, warm-mix flame-retardant SBS composite modified asphalt has been proposed to meet the combined requirements of tunnel pavement construction and fire safety [17,18]. Such composite systems can potentially integrate the advantages of polymer modification, warm-mix construction, and flame-retardant technology. From the perspective of flame-retardant modification, Bonati et al. [19] investigated the ignitability and thermal stability of asphalt binders and mastics for tunnel pavements. Their results showed that aluminium hydroxide markedly improved the limiting oxygen index, while the effectiveness of flame-retardant fillers was closely related to their decomposition temperature and physical characteristics. Sheng et al. [20] developed an environmentally friendly flame-retardant composite for asphalt binders used in tunnel pavements. They reported that the composite system could improve the low-flammability behavior of asphalt without seriously compromising mixture performance. Hull et al. [21] clarified the flame-retardant action of mineral fillers, demonstrating that metal hydroxides can suppress combustion through endothermic decomposition, dilution of the gas phase, and enhancement of the heat capacity of combustion residues. Wan et al. [22] developed an Evotherm pre-wet modified aluminum hydroxide flame-retardant system, which improved the dispersion of flame-retardant particles in asphalt and enhanced the oxygen index. Liu et al. [23] investigated the rheological properties and micromechanism of warm-mix flame-retardant asphalt containing Sasobit and Aluminum Trihydrate/Organic Modified Montmorillonite, and reported that the flame-retardant dosage improved high-temperature rheological properties but adversely affected low-temperature performance. These studies demonstrate that polymer modification, warm-mix technology, and flame-retardant modification can be integrated to improve the comprehensive performance of asphalt binders for tunnel pavements.

However, most existing studies have focused on conventional road performance or limited flame-retardant indicators, such as flash point and oxygen index [24,25,26]. The combined effects of different modifiers on limiting oxygen index, smoke density rating, rheological properties, aging behavior, low-temperature cracking resistance, and microscopic mechanisms remain insufficiently clarified. In particular, the thermal decomposition behavior and functional-group characteristics of fast-melting warm-mix flame-retardant composite modified asphalt require further investigation. Therefore, a systematic evaluation of its flame-retardant, smoke-suppression, rheological, aging, and microscopic properties is necessary for promoting its application in tunnel asphalt pavements.

To address these gaps, this study systematically investigates the flame-retardant performance, rheological behavior, aging sensitivity, low-temperature cracking resistance, and microscopic mechanism of fast-melting warm-mix flame-retardant composite modified asphalt binders. Different modified asphalt binders were prepared and evaluated using limiting oxygen index and smoke density tests to characterize their flame-retardant and smoke-suppression performance. Frequency sweep and bending beam rheometer tests were conducted to analyze their viscoelastic response, aging characteristics, and low-temperature performance. In addition, thermogravimetric (TG) analysis and Fourier-transform infrared spectroscopy (FTIR) were used to reveal the thermal decomposition behavior and functional-group characteristics of the modified binders. This work aims to clarify the performance balance and modification mechanism of fast-melting warm-mix flame-retardant composite modified asphalt, providing technical support for its application in tunnel asphalt pavements.

## 2. Materials and Methods

### 2.1. Materials

Pen 70 paving petroleum asphalt was used as the base binder in this study, and its basic properties are summarized in Table 1. To investigate the effects of the fast-melting warm-mix flame-retardant composite modifier on the flame-retardant and rheological properties of asphalt, three polymer modifiers, one warm-mix additive, and one flame retardant were selected. The polymer modifiers included a conventional styrene-butadiene-styrene (SBS) modifier, a fast-melting SBS modifier (SBS-T), and a fast-melting warm-mix flame-retardant composite modifier (SBS-WZ). SBS-WZ, supplied by Guolu Gaoke (Beijing) Engineering Technology Research Institute Co., Ltd., Beijing, China, is a fast-melting warm-mix flame-retardant composite modifier prepared by physically blending a fast-melting SBS modifier with warm-mix and flame-retardant components. It appears as light-green granules. In this study, SBS-WZ represents an integrated modification route combining fast-melting, warm-mix, and flame-retardant functions. Sasobit was used as the warm-mix additive. FR02, supplied by Guolu Gaoke (Beijing) Engineering Technology Research Institute Co., Ltd., Beijing, China, was used as a composite smoke-suppressing flame retardant. It appears as a uniform white powder with a fineness greater than 40 mesh. Its main function is to improve the flame-retardant and smoke-suppression performance of asphalt binders. The technical properties of these modifiers are shown in Table 2.

### 2.2. Preparation of Asphalt Binders

#### 2.2.1. Preparation of Modified Asphalt Binders

Based on preliminary formulation optimization and engineering application requirements, the dosages of Sasobit, SBS, SBS-T, SBS-WZ, and FR02 were determined as 1.5 wt.%, 4 wt.%, 4 wt.%, 13 wt.%, and 10 wt.%, respectively. Seven modified asphalt binders were prepared in this study, and their formulations are listed in Table 3. All modifier dosages were calculated by weight of the base asphalt.

All modified asphalt binders were prepared using a wet modification process. First, a specified mass of 70# base asphalt was heated in an oven at 150 °C until it was completely melted and reached a fluid state. Subsequently, the designed amounts of SBS, SBS-T, SBS-WZ, Sasobit, and FR02 were weighed and gradually added to the corresponding mass of base asphalt. During addition, the binders were pre-mixed using a glass rod to initially disperse the modifiers and prevent local agglomeration.

After pre-mixing, the container holding the asphalt blend was placed under a FLUKO FM300 (manufactured by FLUKO Technology Development Shanghai Co., Ltd., Shanghai, China) high-speed shear mixer for shearing and dispersion. The shearing speed was controlled at 4500 ± 100 r/min, and the shearing time was 30 min. During shearing, the binder temperature was maintained at 160 ± 10 °C. The temperature was monitored throughout the shearing process and adjusted when necessary to avoid excessive heating and prolonged exposure of the polymer modifiers to high temperatures. Mixing was conducted under ambient laboratory atmosphere without an inert-gas supply. The mixer did not provide real-time torque acquisition; therefore, torque was not used as a process-control parameter. After shearing, the modified asphalt binder was conditioned in an oven for 60 min to promote sufficient swelling, dispersion, and stabilization of the modifiers in the asphalt matrix. To minimize preparation-induced variability, the modifier-addition sequence, binder mass, heating temperature, shearing speed, shearing duration, and post-shearing conditioning time were kept identical for all formulations. The repeatability evaluated in this study refers to replicate performance tests conducted on binders prepared using this controlled protocol. No independent microscopic dispersion analysis or batch-to-batch preparation test was performed; therefore, quantitative uniformity of modifier dispersion and mixing repeatability cannot be directly established from the present experiments. The resulting binders were used for subsequent flame-retardant, rheological, and microstructural tests.

#### 2.2.2. Aging Procedures

To evaluate the evolution of rheological properties of different modified asphalt binders under aging conditions, the Rolling Thin Film Oven Test (RTFOT) and Pressure Aging Vessel (PAV) tests were used to simulate short-term and long-term aging, respectively.

RTFOT aging was performed using an SYD-0609 rolling thin-film oven (manufactured by Shanghai Changji Geological Instrument Co., Ltd., Shanghai, China). After the modified asphalt binders were heated to a fluid state, 35 ± 0.5 g of binder was poured into a dedicated RTFOT glass bottle. The aging temperature was set at 163 °C, the air flow rate was 4000 ± 200 mL/min, the turntable rotation speed was 15 ± 0.2 r/min, and the aging time was 85 min.

PAV aging was conducted using a PAV-1 pressure aging vessel (manufactured by Beijing Biaoxing Aidu Instrument Co., Ltd., Beijing, China). The RTFOT-aged modified asphalt binders were first heated to a fluid state, and 50 ± 0.5 g of binder was poured into a flat-bottom stainless-steel pan with dimensions of Φ140 mm × 10 mm, producing an asphalt film thickness of approximately 3.2 mm. The pans containing the asphalt samples were then placed in the pressure aging vessel and aged at 100 °C and 2.1 ± 0.1 MPa for 20 h. The aged binders were used for DSR frequency sweep tests and BBR low-temperature creep tests.

### 2.3. Flame-Retardant Performance

#### 2.3.1. Limiting Oxygen Index (LOI)

The LOI test was used to evaluate the flame-retardant performance of different modified asphalt binders [28]. The test was conducted according to the Oxygen Index Method for Combustion Performance using an XZT-100A oxygen index analyzer (Chengde Kecheng Testing Machine Co., Ltd., Chengde, China). The minimum oxygen volume fraction required to sustain continuous combustion of the specimen was determined by adjusting the oxygen/nitrogen mixed gas flow. A higher LOI value indicates better flame-retardant performance. Considering that asphalt is prone to softening, melting, and dripping before combustion, a glass fiber surface mat was used as the carrier substrate for preparing LOI specimens. The glass fiber surface mat was cut into 110 ± 1 mm × 110 ± 1 mm squares, dried at 120 ± 2 °C for 1.0–1.5 h, cooled to room temperature, and weighed. Molten asphalt was then uniformly coated onto the glass fiber mat at a mass equal to 60 times that of the mat. After cooling, the coated mat was cut into strip specimens with dimensions of 100 mm × 5.5 mm. At least three specimens were prepared for each modified asphalt binder for LOI testing.

#### 2.3.2. Smoke Density Rating (SDR)

The SDR was used to evaluate the smoke generation characteristics of different modified asphalt binders during combustion. The test was conducted according to the GB/T 8627-2007 [29], using a JCY-2 smoke density tester for building materials (Nanjing Jiangning Analytical Instrument Co., Ltd., Nanjing, China). Before testing, the instrument was calibrated using filters with optical absorptance values of 25%, 50%, and 75%. During testing, the specimen was horizontally placed on a metal support inside the smoke chamber, with the flame positioned directly beneath the specimen. The propane pressure was set to 276 kPa. Optical absorptance was recorded at 15 s intervals for a total duration of 4 min. For specimen preparation, asphalt was conditioned at 165 ± 5 °C for 1.5–2.0 h until it reached a fluid state and was then poured into a 25 mm × 25 mm × 6 mm mold. After cooling and demolding, the specimens were trimmed to 25.4 ± 0.3 mm × 25.4 ± 0.3 mm × 6.2 ± 0.3 mm. At least three replicate specimens were prepared for each asphalt binder. The maximum smoke density and smoke density rating (SDR) were used to characterize smoke suppression performance; a lower SDR indicates better smoke suppression capability.

#### 2.3.3. Thermogravimetric Analysis (TG)

**Figure 1:**
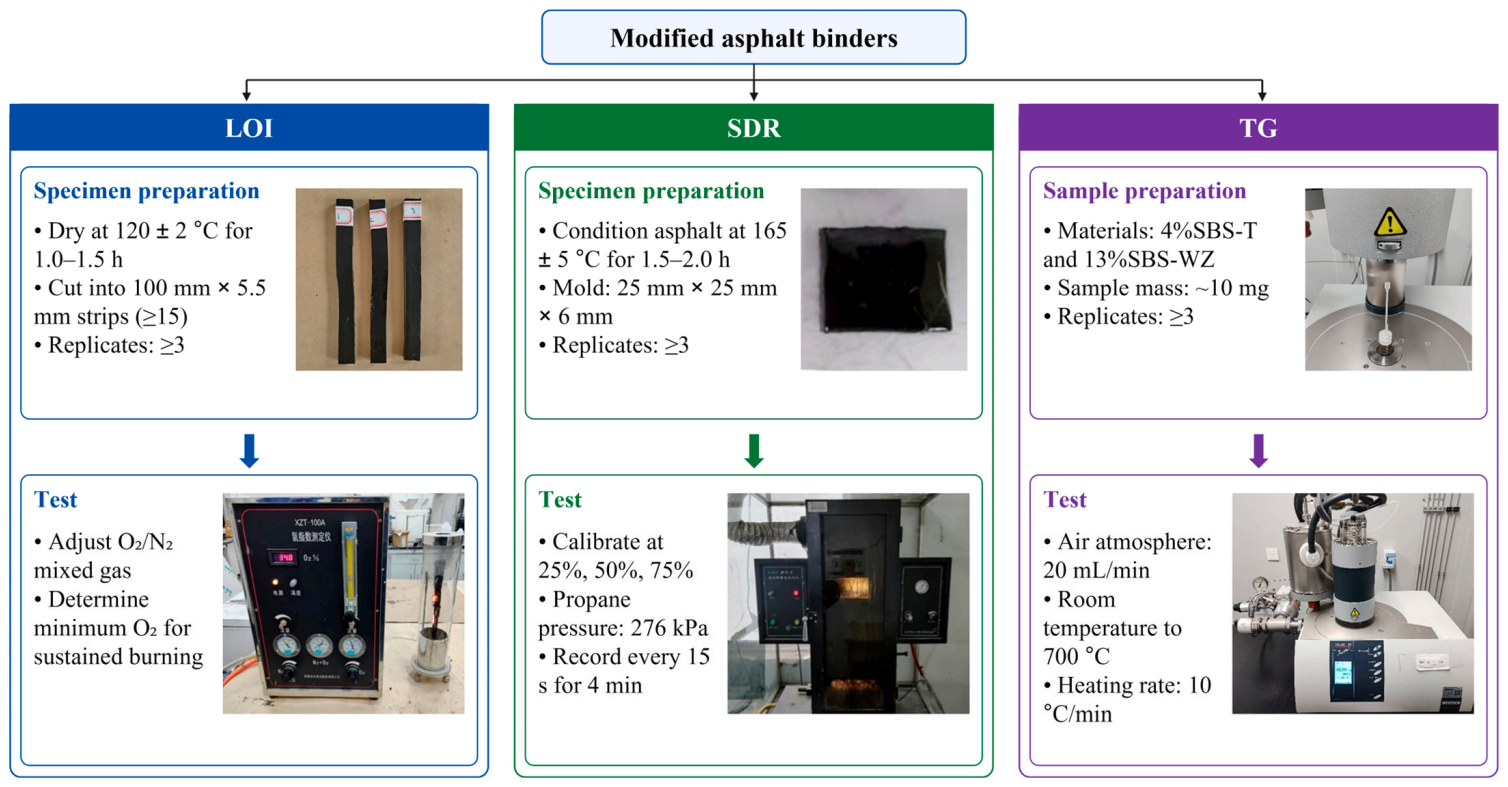
Experimental workflow for flame-retardant performance of modified asphalt binders.

TG was used to evaluate the thermal stability and thermal decomposition behavior of the modified asphalt binders. The tests were conducted using a NETZSCH STA 449 F3 Jupiter simultaneous thermal analyzer (manufactured by NETZSCH Group, Selb, Germany). The 4%SBS-T-modified asphalt and 13%SBS-WZ-modified asphalt were tested. Approximately 10 mg of each sample was heated under an air atmosphere at a gas flow rate of 20 mL/min. The temperature range was from room temperature to 700 °C, and the heating rate was 10 °C/min. Three replicate tests were performed for each asphalt binder. The onset decomposition temperature, final decomposition temperature, and residual mass at 700 °C were analyzed from the TG curves to evaluate the effects of SBS-WZ on the thermal stability and flame-retardant mechanism of asphalt. Figure 1 shows the Experimental workflow for flame-retardant performance evaluation of modified asphalt binders.

### 2.4. Rheological Properties

#### 2.4.1. Master Curves

The intermediate-temperature viscoelastic properties of the modified asphalt binders were evaluated using a dynamic shear rheometer (DSR; Discovery HR-1, TA Instruments, New Castle, DE, USA). The asphalt binder was heated to a fluid state, poured into a silicone mold with a diameter of 8 mm, cooled, demolded, and placed between 8 mm parallel plates for testing. Frequency sweep tests were performed in strain-controlled mode at a strain level of 0.1% to ensure testing within the linear viscoelastic range. The test temperatures were 15 °C, 25 °C, and 35 °C, and the angular frequency range was 0.1–100 rad/s. Unaged, RTFOT-aged, and PAV-aged binders were tested. Three replicate specimens were prepared for each condition, and the average values were used for analysis.

Based on the time–temperature superposition principle, master curves of complex modulus and phase angle were constructed using 25 °C as the reference temperature. The Williams–Landel–Ferry (WLF) equation was used to calculate temperature shift factors. The complex modulus master curve was fitted using the Christensen–Anderson–Marasteanu (CAM) model to characterize the viscoelastic response and aging sensitivity of different modified asphalt binders over a wide frequency range. In the present study, the CAM model was used only to obtain continuous representations of the complex-modulus master curves. Its fitting parameters were not treated as independent rheological performance indices.

#### 2.4.2. Low-Temperature Cracking Resistance

The low-temperature cracking resistance of the modified asphalt binders was evaluated using a bending beam rheometer (BBR). Beam specimens of unaged and long-term-aged binders were prepared with dimensions of 102 mm × 12.5 mm × 6.25 mm. The BBR tests were conducted using a Model 1800 bending beam rheometer supplied by Earth Products China Ltd. (Hong Kong, China) in accordance with ASTM D6648. The test temperatures were set at −12 °C, −18 °C, and −24 °C. During testing, the creep response of the asphalt beam under constant loading was recorded, and the creep stiffness S (60 s) and creep rate m (60 s) at 60 s were calculated. A lower S (60 s) indicates lower low-temperature stiffness, whereas a higher m (60 s) indicates stronger stress relaxation capability. Three replicate beam specimens were tested for each binder, temperature, and aging condition.

#### 2.4.3. Fourier Transform Infrared Spectroscopy (FTIR)

**Figure 2:**
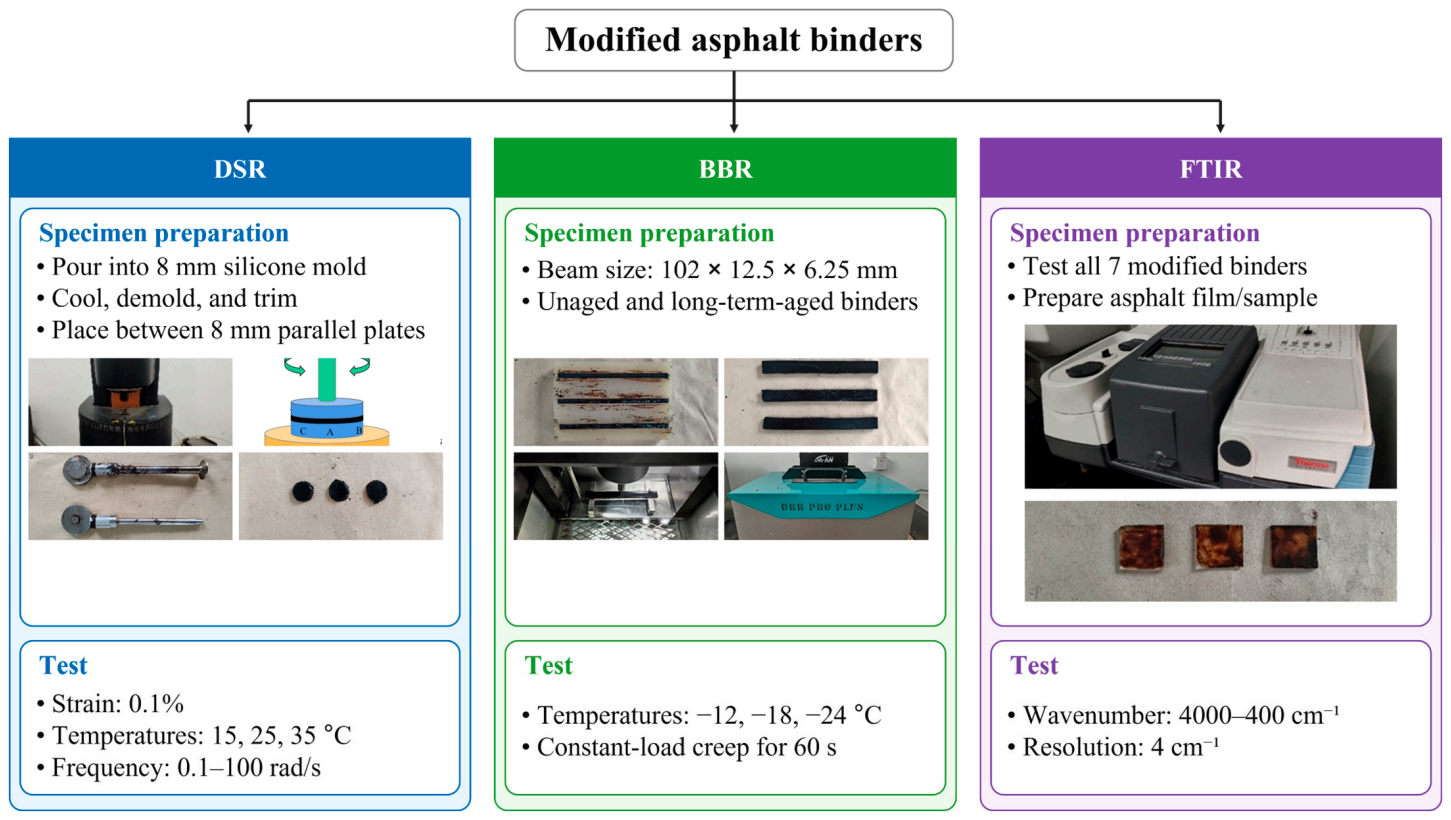
Experimental workflow for rheological properties of modified asphalt binders.

FTIR was used to characterize the effects of different modifiers on the chemical structure of asphalt. The tests were performed using a Nicolet Continuum FTIR spectrometer (Thermo Fisher Scientific, Waltham, MA, USA) on the seven modified asphalt binders to identify changes in characteristic functional groups before and after modification and to reveal the interaction mechanisms between SBS-T, Sasobit, FR02, SBS-WZ, and asphalt. Spectra were collected over the wavenumber range of 4000–400 cm^−1^ at a resolution of 4 cm^−1^, with 32 scans for each spectrum. Figure 2 shows the experimental workflow for rheological properties of modified asphalt binders. In the present study, TG and FTIR were selected as complementary techniques to characterize thermal decomposition behavior and functional-group characteristics, respectively. 

## 3. Flame-Retardant Performances

### 3.1. Limiting Oxygen Index and Smoke Density Rating

**Figure 3:**
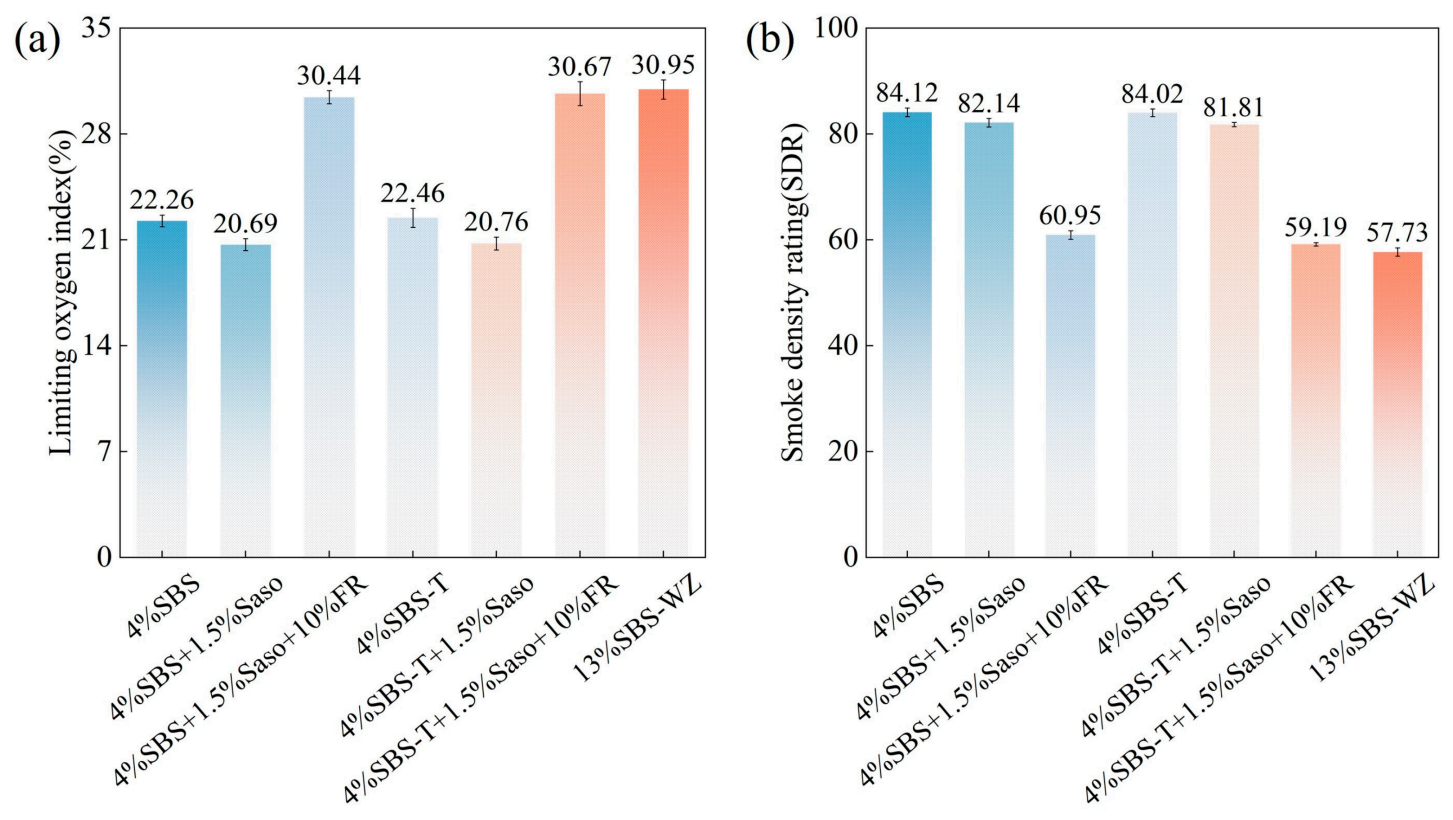
Flame-retardant performance of different modified asphalt binders: (**a**) limiting oxygen index; (**b**) smoke density rating.

Figure 3a presents the LOI results of different modified asphalt binders. It can be seen that 13%SBS-WZ exhibited the highest LOI values, reaching 30.95%, whereas 4%SBS + 1.5%Sasobit showed the lowest values, with a value of only 20.69%. Sasobit mainly consists of long-chain aliphatic waxes. Although it reduces binder viscosity at elevated temperatures, its hydrocarbon-rich structure may contribute to the generation of combustible volatile products during heating, thereby lowering the oxygen concentration required to sustain combustion. Because the gaseous decomposition products were not directly analyzed, this explanation is proposed as a mechanism consistent with the measured LOI trend rather than as direct experimental proof. The higher LOI of 13%SBS-WZ directly indicates its greater resistance to sustained combustion under the present test conditions. However, the internal polymer-network structure was not directly characterized in this study; therefore, the improvement cannot be conclusively attributed to the formation of a more stable polymer network. The LOI values of 4%SBS and 4%SBS-T were 22.26% and 22.46%, respectively, indicating that the improvement effect of fast-melting SBS on the flame-retardancy of asphalt binders is not significant. After the addition of 1.5% Sasobit, the LOI values of 4%SBS and 4%SBS-T decreased to 20.69% and 20.76%, corresponding to reductions of 7.05% and 7.57%, respectively. In contrast, FR02 increased the LOI values of both warm-mix systems by more than 47%. These reductions indicate a substantial improvement in smoke suppression, which is particularly relevant to tunnel visibility and evacuation safety [30,31]. In contrast, the incorporation of FR02 markedly increased the LOI values of the modified asphalt binders, and the improvement ratios for both modified asphalt binder systems exceeded 47%. The increase in LOI may be associated with condensed-phase flame-retardant effects, including heat absorption and enhanced retention of thermally stable residues [32]. However, the morphology, continuity, and protective effectiveness of the combustion residue were not directly characterized in this study. Therefore, formation of a continuous protective char layer cannot be regarded as experimentally verified.

As shown in Figure 3b, the addition of Sasobit decreased the SDR of both modified asphalt binders slightly. Specifically, for the 4%SBS, the SDR decreased from 84.10 to 82.10, representing a reduction of 2.38%; for the 4%SBS-T, the SDR decreased from 84.00 to 81.80, corresponding to a reduction of 2.62%. This indicates that Sasobit can slightly reduce the SDR of asphalt binders; however, the values still fail to meet the requirement of SDR ≤ 75 for extra-long tunnels [33]. In contrast, the incorporation of FR02 markedly reduced the smoke density rating of the warm-mix modified asphalt binders. The SDR values of 4%SBS + 1.5%Sasobit + 10%FR02 and 4%SBS-T + 1.5%Sasobit + 10%FR02 decreased to 60.90 and 59.20, respectively. LOI characterizes the oxygen concentration required to sustain combustion, whereas SDR characterizes the optical density of the smoke generated during combustion or decomposition. FR02 may suppress combustion through heat absorption, delayed release of combustible volatile compounds, and increased condensed-phase retention. The corresponding reduction in combustion intensity and volatile release may decrease the formation of aromatic and carbonaceous smoke precursors, thereby lowering the SDR [34].

### 3.2. TG Results

**Figure 4:**
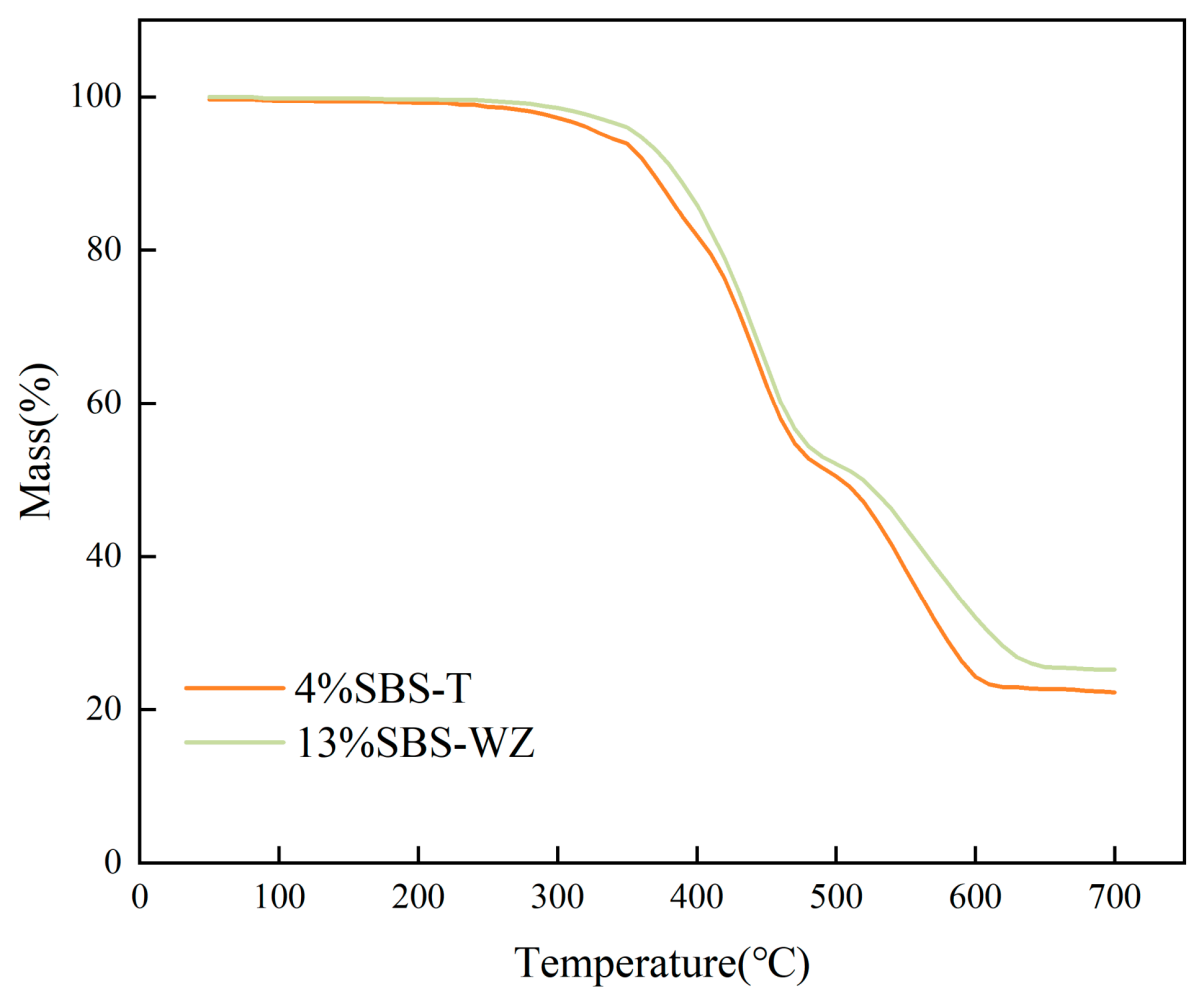
TG spectra of different modified asphalt binders.

As shown in Figure 4, both 4%SBS-T and 13%SBS-WZ exhibit distinct staged thermal weight-loss characteristics. Compared with 4%SBS-T, 13%SBS-WZ shows a higher initial decomposition temperature, final decomposition temperature, and residual mass at 700 °C, indicating its superior thermal stability and char-forming capacity at high temperature. In the range of 0–300 °C, the mass loss of both modified asphalt binders is relatively limited, suggesting that they remain thermally stable within this temperature range. As the temperature further increases, the samples enter the main thermal decomposition stage. The onset decomposition temperature increased from 374.9 °C for 4%SBS-T to 392.1 °C for 13%SBS-WZ. The increase of 17.2 °C indicates delayed initiation of rapid thermal decomposition, suggesting improved thermal stability within this two-binder comparison. During heating, FR02 can preferentially decompose and absorb heat, while releasing non-flammable gases to dilute combustible volatiles, thereby delaying the thermal decomposition of the asphalt matrix [35].

For 4%SBS-T, the weight-loss rate increases markedly within 374.9–461.7 °C, which is mainly associated with the volatilization of light components and the decomposition of resins and asphaltenes. In the range of 461.7–598.2 °C, various residual components continue to participate in the decomposition process. When the temperature exceeds 598.2 °C, the weight-loss rate gradually decreases, indicating that most decomposable components have been consumed. 13%SBS-WZ shows a similar thermal decomposition behavior within 392.1–626.3 °C; however, its decomposition range shifts toward higher temperatures, further confirming that FR02 enhances the thermal stability of the asphalt system. Moreover, the residual mass of 13%SBS-WZ at 700 °C is higher than that of 4%SBS-T, indicating the formation of a more stable char residue at high temperature. This char layer can act as a barrier to heat transfer and the release of combustible volatiles, thereby suppressing further thermal decomposition and combustion propagation [36]. These results are consistent with previous studies reporting that mineral and composite flame retardants can delay asphalt decomposition, increase residual mass, improve the limiting oxygen index, and reduce smoke generation through combined gas-phase and condensed-phase effects [19,20,21]. However, the present study further shows that the improvement in fire-related performance is accompanied by increased complex modulus and creep stiffness and reduced creep rate, indicating a trade-off between flame retardancy and low-temperature flexibility. Compared with the separately blended FR02 systems, SBS-WZ provided a more balanced overall response, although its low-temperature performance remained inferior to that of conventional SBS-modified binders.

## 4. Rheological Properties of Flame-Retardant Asphalt Binders

### 4.1. Master Curves Results

**Figure 5:**
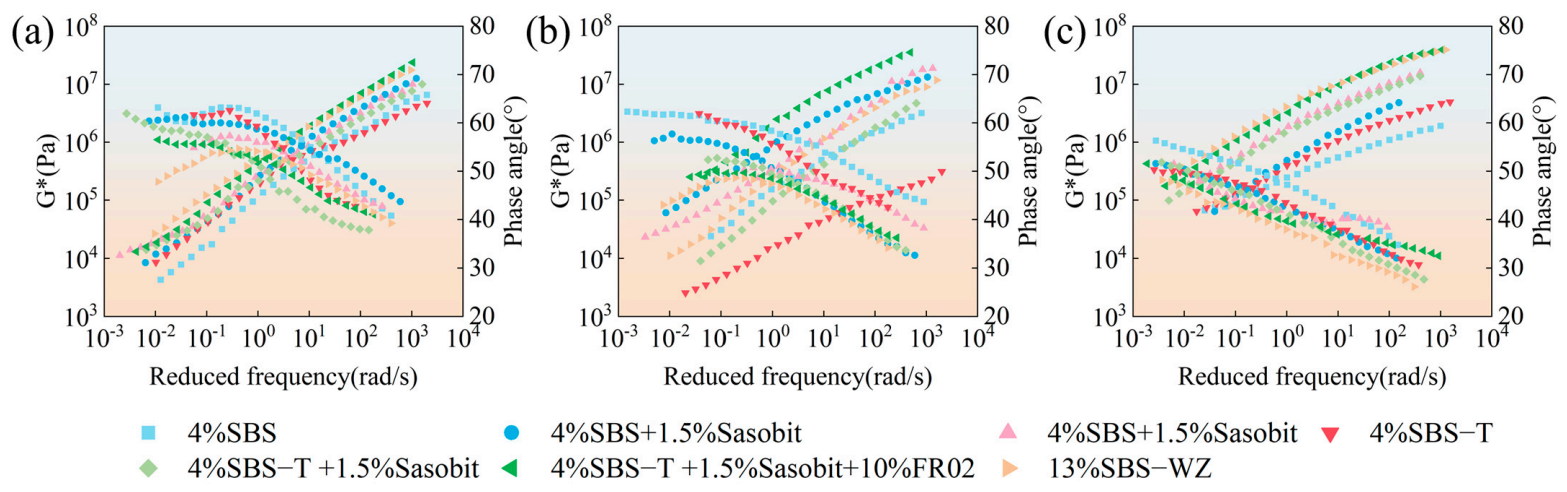
Master curves of complex modulus and phase angle of modified asphalt binders under different aging conditions: (**a**) unaged, (**b**) RTFOT-aged, and (**c**) PAV-aged.

As illustrated in Figure 5, the complex modulus of all asphalt binders increases with increasing reduced frequency, indicating a pronounced frequency-dependent rheological response. This trend reflects the high-frequency stiffening behavior of asphalt binders, suggesting that the materials exhibit higher resistance to deformation. At the same loading frequency, the incorporation of Sasobit increases the complex modulus of the modified asphalt binders, demonstrating that the warm-mix additive contributes positively to stiffness enhancement [37]. In addition, under different aging conditions, the 13%SBS-WZ exhibits a relatively high complex modulus in the low-frequency region, indicating that the SBS-WZ can effectively improve the stiffness and structural strength of the binder. Aging generally increases the complex modulus of modified asphalt binders. During aging, part of the lighter components is lost, while oxygen-containing polar products and the relative asphaltene content increase. These changes strengthen intermolecular association and reduce viscous flow, resulting in a higher complex modulus and a lower phase angle. The longer oxidative exposure during PAV aging therefore produces a more pronounced hardening effect than RTFOT aging [38]. However, an increase in complex modulus does not necessarily indicate improved pavement performance. Excessive stiffness may reduce the flexibility of asphalt binders and increase their susceptibility to brittle cracking under heavy traffic loading or low-temperature service conditions. Moreover, as the aging degree increases, the slope of the complex modulus master curve gradually decreases, suggesting that aging weakens the rheological differences in the binders over different frequency ranges and reduces their temperature sensitivity.

The phase angle of different modified asphalt binders under various aging conditions generally decreases with increasing reduced frequency. At the same loading frequency, the Sasobit-modified binders exhibit lower phase angle values than the corresponding binders without Sasobit, suggesting a higher elastic component and improved resistance to deformation [38]. In the low-frequency region, the 13%SBS-WZ shows the lowest phase angle among the investigated binders under different aging conditions, indicating its superior deformation resistance and more pronounced elastic response. Compared with RTFOT-aged binders, the master curves of PAV-aged binders are located at lower levels, implying that long-term oxidative aging further enhances the elastic-dominated behavior of the binders and reduces their viscous energy dissipation capacity [39]. In addition, plateau regions or peak values can be observed in the phase angle master curves of some modified asphalt binders. With increasing aging degree, these plateau regions or peaks gradually become less distinct or even disappear, suggesting that aging alters the compositional balance of asphalt binders and weakens the modifier–asphalt interaction. In this study, the CAM and WLF formulations were used mainly to construct the master curves, and their fitting parameters were not treated as independent performance indices. Therefore, the rheological comparison is based primarily on the relative positions and evolution of the master curves. A detailed parameter-level sensitivity analysis should be considered in future work.

### 4.2. Low-Temperature Cracking Resistance Results

**Figure 6:**
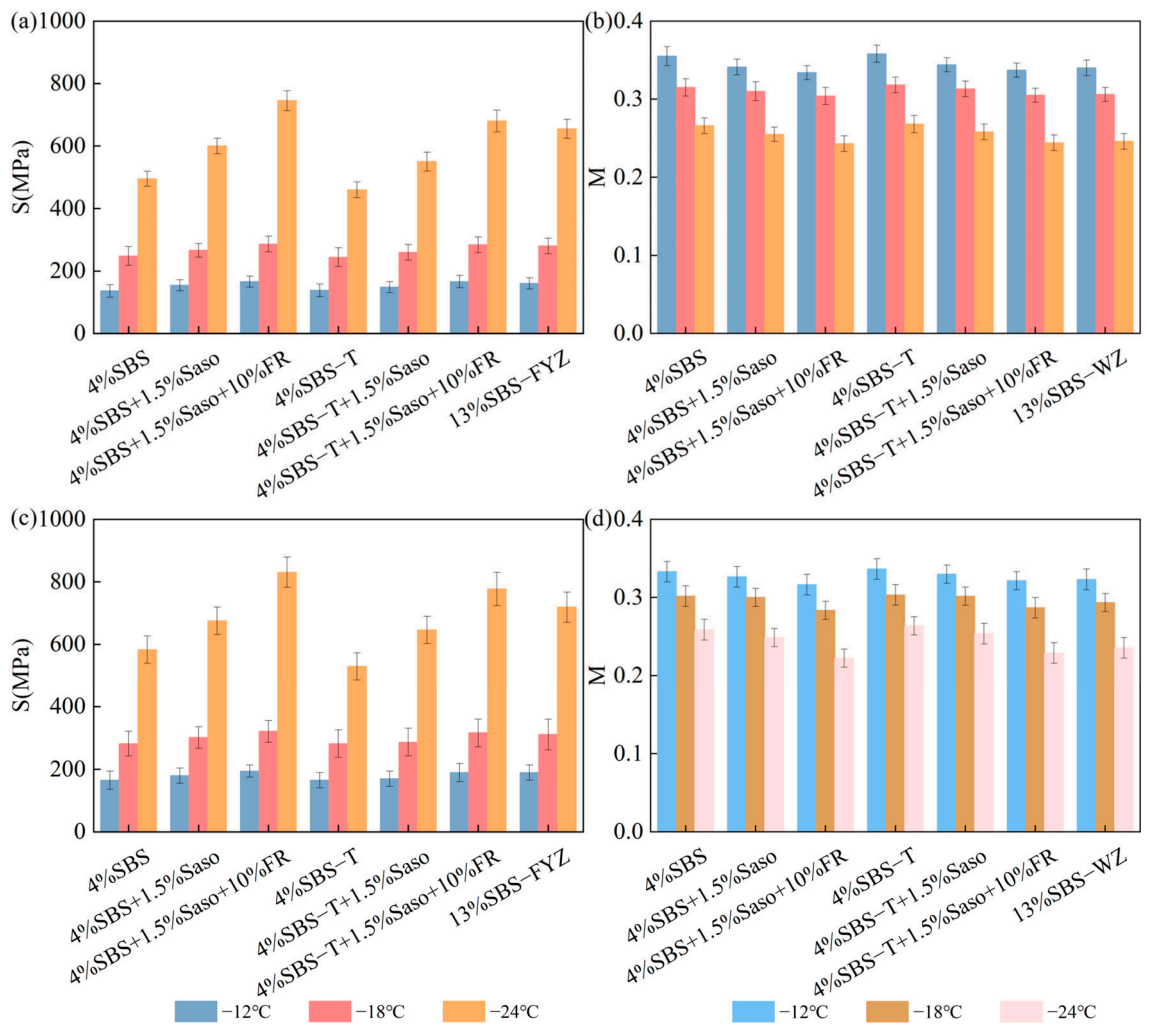
Low-temperature properties of asphalt binders: (**a**) stiffness modulus of the unaged binder; (**b**) creep rate of the unaged binder; (**c**) stiffness modulus of the PAV-aged binder; (**d**) creep rate of PAV-aged binder.

As shown in Figure 6, the S and M of the modified asphalt binders under different aging conditions exhibit clear temperature dependence. As the test temperature decreases from (−12 °C to −24 °C), the S values of all asphalt binders increase markedly, whereas the M values gradually decrease. This indicates that low temperature restricts the mobility of asphalt molecular chains, leading to increased material stiffness and reduced stress relaxation capacity. Therefore, a lower S value and a higher M value generally correspond to better low-temperature deformation compatibility and cracking resistance. In terms of material composition, 4%SBS and 4%SBS-T exhibit relatively low stiffness modulus and high creep rate, indicating that long-term aging further intensifies asphalt hardening and embrittlement and reduces its low-temperature stress relaxation capacity. This behavior can be mainly attributed to the thermoplastic elastomeric structure of SBS-type modifiers. The soft segments improve the deformation capacity of asphalt at low temperature, whereas the hard segments help maintain structural stability [40]. After the incorporation of 1.5% Sasobit, the S values of the SBS- and SBS-T-modified asphalt binders increase slightly, while the M values decrease. This response can be related to the crystallization of long-chain wax molecules after cooling. The resulting rigid crystalline structure restricts molecular rearrangement and stress relaxation, thereby increasing creep stiffness and reducing the m-value at low temperatures.

With the further addition of 10% FR02, the stiffness modulus of the modified asphalt binders increases markedly, while the creep rate decreases markedly. Among them, the 4%SBS + 1.5%Sasobit + 10%FR02 system exhibits the highest S value and relatively lowest M value, indicating the most pronounced low-temperature hardening and a greater adverse effect on low-temperature cracking resistance. The 4%SBS-T + 1.5%Sasobit + 10%FR02 system shows a similar trend, although its low-temperature hardening degree is slightly lower than that of the SBS-based composite system. In contrast, the S and M values of the 13%SBS-WZ remain at an intermediate level, suggesting that although it increases the low-temperature stiffness of asphalt, its adverse effect on low-temperature performance is weaker than that of the FR02 composite modified systems. According to the Superpave specification for PAV-aged asphalt binders, the creep stiffness at 60 s should satisfy S ≤ 300 MPa, while the creep rate should satisfy m ≥ 0.300. The specification comparison is therefore based primarily on the PAV-aged results rather than the unaged results.

Compared with the unaged samples, the PAV-aged binders show higher S values and lower M values at the same temperature, indicating that long-term aging intensifies asphalt hardening and reduces its low-temperature stress relaxation capacity. At −12 °C, the S-values of the PAV-aged binders were approximately 160–190 MPa, while their m-values ranged from approximately 0.315 to 0.335. Therefore, all seven binders satisfied both Superpave requirements at this temperature. This may be attributed to the loss of light components. The increase in polar oxidation products and asphaltene content during aging, and the resulting enhancement of intermolecular interactions [41]. Consequently, the asphalt system gradually transforms from a flexible viscoelastic state to a high-stiffness brittle state. The Sasobit-induced increase in stiffness agrees with previous studies attributing this response to wax crystallization and restricted molecular mobility [9,10,34]. The accompanying reduction in low-temperature stress relaxation is also consistent with reports that warm-mix additives may adversely affect cracking resistance. Similarly, the increase in LOI and reduction in SDR after FR02 incorporation agree with previous findings on mineral and composite flame retardants [17,18,19,20,21]. However, the present results further show that these fire-related benefits are accompanied by increased creep stiffness and reduced m-values. This adverse effect was more pronounced for the separately blended FR02 systems than for SBS-WZ, although conventional SBS and SBS-T still provided better low-temperature flexibility.

### 4.3. FTIR Results

**Figure 7:**
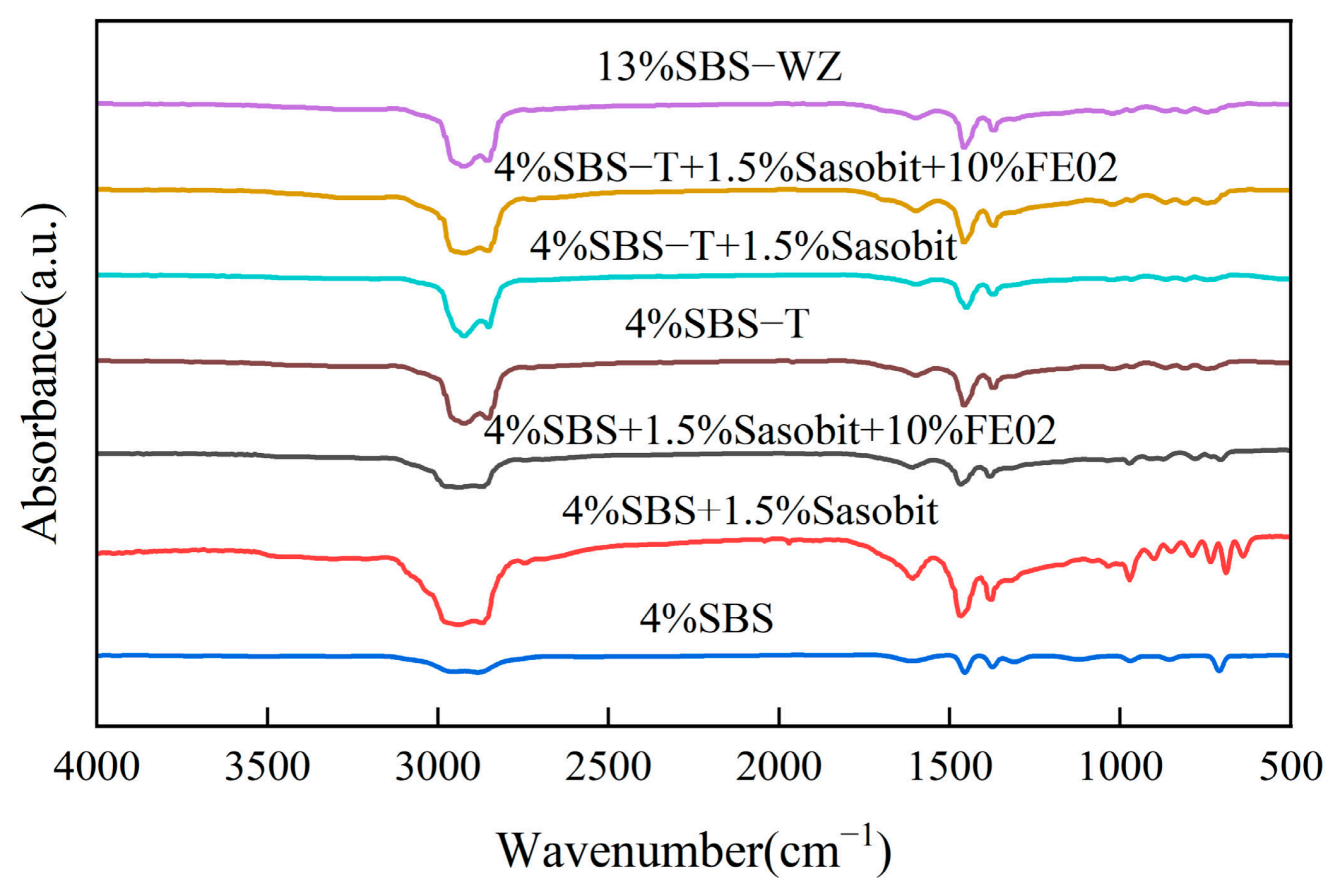
FTIR spectra of different modified asphalt binders.

As shown in Figure 7, all modified asphalt binders exhibit distinct absorption bands in the range of 2930–2850 cm^−1^, which are mainly assigned to the stretching vibration of methylene groups -CH2-. After the incorporation of Sasobit, FR02, and SBS-WZ, no obvious shift is observed in the positions of the main characteristic peaks. Oxidative aging of asphalt is commonly associated with increases in carbonyl-related absorption near 1700 cm^−1^ and sulfoxide-related absorption near 1030 cm^−1^. These oxygen-containing groups reflect oxidation of susceptible hydrocarbon components and are often associated with increased polarity and binder hardening. This result indicates that no major new functional groups were detected within the resolution of the FTIR measurements. It suggests that physical blending and intermolecular interactions may dominate the modification process. However, unchanged peak positions alone are insufficient to exclude weak chemical reactions, hydrogen bonding, interfacial interactions, or overlapping absorption bands [39].

After adding 1.5% Sasobit, the absorption peaks of both SBS- and SBS-T-modified asphalt binders increase in the range of 2930–2800 cm^−1^ with a more pronounced increase observed for the SBS-modified asphalt binder. This may be attributed to the introduction of long-chain aliphatic structures from Sasobit, which increases the relative content of -CH2- and -CH3- groups in the asphalt system [42]. Meanwhile, Sasobit may also affect the interaction between the polymer modifier and the saturated fractions in the base asphalt, thereby changing the intensity of the characteristic absorption peaks.

Furthermore, noticeable differences are observed between the warm-mix flame-retardant and the fast-melting modified asphalt. This suggests that different modifiers have different effects on the functional-group environment and intermolecular interactions within the asphalt binder. In particular, 13%SBS-WZ shows a characteristic absorption peak at approximately 1376 cm^−1^, which can be attributed to the bending vibration of methyl groups -CH^3-^ in the molecular chain of the SBS-WZ copolymer [43]. Overall, the addition of Sasobit, FR02, and SBS-WZ mainly changes the peak intensity and local spectral shape, while no significant shift in the major characteristic peaks is observed. These observations are consistent with a modification process dominated by physical blending and component interactions. Nevertheless, FTIR alone cannot fully distinguish physical interactions from weak chemical interactions, and additional techniques would be required to verify the interaction mechanism.

## 5. Conclusions

In this study, the flame-retardant performance, rheological properties, and low-temperature performance of different modified asphalt binders were systematically investigated. Furthermore, microscopic characterization was conducted to reveal the flame-retardant mechanisms. The main conclusions are summarized as follows:

1. Sasobit slightly reduced the flame-retardant performance of the modified asphalt binders, whereas FR02 markedly increased the limiting oxygen index and reduced the smoke density rating. Among all formulations, 13%SBS-WZ exhibited the best fire-related performance under the present test conditions, demonstrating the effectiveness of integrating fast-melting, warm-mix, and flame-retardant technologies.
2. The DSR results showed that 13%SBS-WZ exhibited a relatively high complex modulus and a low phase angle, indicating increased stiffness and a more elastic-dominated response, while PAV aging produced more pronounced rheological changes than RTFOT aging. However, the increased stiffness did not necessarily represent an overall performance benefit because it was accompanied by reduced deformation compatibility at low temperatures.
3. The BBR results showed that 4%SBS and 4%SBS-T exhibited the best low-temperature flexibility, whereas Sasobit and especially FR02 increased creep stiffness and reduced creep rate. Although SBS-WZ performed better than the separately blended SBS/SBS-T–Sasobit–FR02 systems, its low-temperature performance remained inferior to that of conventional SBS binders, indicating a trade-off between improved flame retardancy and low-temperature cracking resistance.
4. SBS-WZ exhibited higher thermal stability and greater residue-forming potential than 4%SBS-T, while FTIR suggested that the modification was dominated by physical blending and component interactions. Combined with the DSR and BBR results, these findings indicate that the improved fire-related performance of SBS-WZ is accompanied by increased binder stiffness and reduced molecular mobility. This study provides experimental evidence for understanding the balance between flame retardancy and rheological performance in fast-melting warm-mix flame-retardant modified asphalt binders, offering guidance for the design of multifunctional asphalt materials for tunnel pavements.

This study systematically evaluated the flame-retardant and smoke-suppression performance, aging-dependent rheological behavior, low-temperature cracking resistance, thermal stability, and functional-group characteristics of fast-melting warm-mix flame-retardant modified asphalt binders. However, the investigation was limited to the binder scale and fixed modifier dosages. The TG analysis included only 4%SBS-T and 13%SBS-WZ, while CAM parameters, quantitative FTIR indices, and statistical significance were not comprehensively analyzed. Modifier dispersion, batch-to-batch repeatability, long-term flame-retardant durability, mixture-scale performance, and field applicability also remain to be verified. Future studies should therefore focus on dosage optimization, broader thermal and microscopic characterization, quantitative CAM and FTIR analyses, statistically designed experiments, asphalt-mixture performance tests, and field trials under actual tunnel conditions.

## Figures and Tables

**Table 1:** Technical properties of base binder.

Property	Units	Test Results	Standard
Penetration at 25 °C	0.1 mm	69.8	T0604-2011 [27]
Softening point	°C	51.7	T0606-2011 [27]
Ductility at 15 °C	cm	>100	T0605-2011 [27]
Brookfield viscosity at 135 °C	Pa·s	0.63	T0625-2011 [27]

**Table 2:** Basic technical properties of SBS, SBS-T, SBS-WZ, and Sasobit.

Modifier	Property	Test Results	Units
SBS	Block ratio (S/B)	30/70	-
	Oil content	0.70	%
	Volatile matter content	1.00	%
	Total ash content	0.20	%
SBS-T	Single-particle mass	0.21	g
	Ash content	0.48	%
	Melt flow rate	2.39	g/10 min
SBS-WZ	Single-particle mass	0.20	g
	Melt flow rate	2.40	g/10 min
Sasobit	Melting point	100	°C
	Flash point	290	°C
	Viscosity at 135 °C	5.47 × 10^−3^	Pa·s
	Viscosity at 150 °C	3.26 × 10^−3^	Pa·s
	Penetration at 25 °C	1	0.1 mm
	Penetration at 60 °C	8	0.1 mm

**Table 3:** Formulations of different modified asphalt binders.

Modified Asphalt Binder	SBS (wt.%)	SBS-T (wt.%)	SBS-WZ (wt.%)	Sasobit (wt.%)	FR02 (wt.%)
SBS	4	0	0	0	0
SBS-S	4	0	0	1.5	0
SBS-SF	4	0	0	1.5	10
SBS-T	0	4	0	0	0
SBS-TS	0	4	0	1.5	0
SBS-TSF	0	4	0	1.5	10
SBS-WZ	0	0	13	0	0

Note: S and F denote Sasobit and FR02, respectively.

## Data Availability

The original contributions presented in this study are included in the article. Further inquiries can be directed to the corresponding authors.
